# *Caenorhabditis elegans* as a valuable model for the study of anthelmintic pharmacodynamics and drug-drug interactions: The case of ivermectin and eprinomectin

**DOI:** 10.3389/fphar.2022.984905

**Published:** 2022-10-19

**Authors:** Gonzalo Suárez, Ignacio Alcántara, Gustavo Salinas

**Affiliations:** ^1^ Unidad de Farmacología y Terapéutica, Departamento Hospital y Clínicas Veterinarias, Facultad de Veterinaria, Universidad de la República, Montevideo, Uruguay; ^2^ Unidad de Bioestadística, Departamento de Salud Pública Veterinaria, Facultad de Veterinaria, Universidad de la República, Montevideo, Uruguay; ^3^ Worm Biology Laboratory, Institut Pasteur de Montevideo, Montevideo, Uruguay; ^4^ Departamento de Biociencias, Facultad de Química, Universidad de la República, Montevideo, Uruguay

**Keywords:** drug combination, antiparasitic resistance, EC50, pharmacology, synergism, nematode, helminth

## Abstract

*Caenorhabditis elegans* is a free-living nematode that has been validated for anthelmintic drug screening. However, this model has not been used to address anthelmintic dose-response-time and drug-drug interactions through matrix array methodology. Eprinomectin (EPM) and Ivermectin (IVM) are macrocyclic lactones widely used as anthelmintics. Despite being very similar, EPM and IVM are combined in commercial formulations or mixed by farmers, under the assumption that the combination would increase their efficacy. However, there is no data reported on the pharmacological evaluation of the combination of both drugs. In this study, we assessed the pharmacodynamics and drug-drug interactions of these two anthelmintic drugs. Since the action of these drugs causes worm paralysis, we used an infrared motility assay to measure EPM and IVM effects on worm movement over time. The results showed that EPM was slightly more potent than IVM, that drug potency increased with drug time exposure, and that once paralyzed, worms did not recover. Different EPM/IVM concentration ratios were used and synergy and combination sensitivity scores were determined at different exposure times, applying Highest Single Agent (HSA), Loewe additivity, Bliss and Zero Interaction Potency (ZIP) models. The results clearly indicate that there is neither synergy nor antagonism between both macrocyclic lactones. This study shows that it is more relevant to prioritize the exposure time of each individual drug than to combine them to improve their effects. The results highlight the utility of *C. elegans* to address pharmacodynamics studies, particularly for drug-drug interactions. Models *in vitro* can be integrated to facilitate preclinical and clinical translational studies and help researchers to understand drug-drug interactions and achieve rational therapeutic regimes.

## Introduction

Drug-drug interactions may alter Pharmacokinetic-Pharmacodynamic (PK/PD) relationship. When combining two or more drugs, the main objective is to achieve positive interaction effects, that is the beneficial effect of the combined drugs compared to each one individually (Tang et al., 2015; Duarte and Vale, 2022). The concepts of synergy and antagonism have accepted definitions, they represent, respectively, greater or lesser effects for the combined drugs than the simple additive effect expected from each drug individually (Foucquier and Guedj, 2015). The altered effect is reflected by the changes in concentrations and/or exposure time in the biophase of the drugs.

Parasitic worms (helminths) constitute a prevalent sanitary and economic problem, particularly for developing countries (Molento et al., 2011). Helminth infections are the causative agents of WHO-categorized neglected diseases, and infect livestock and crops. Helminths are responsible for huge economic losses in animal breeding. Massive pharmacological treatment of livestock against helminthiasis has led producers to face the accelerated spread of anthelmintic resistance to all known classes of anthelmintics (Sangster et al., 2018). In order to delay resistance and treat more effectively helminth infections, the administration of combinations of anthelmintics with a similar spectrum of activity is used as a treatment strategy (Geary et al., 2012; Suarez et al., 2014; Lanusse et al., 2015), most frequently without previous assessment of the effect of synergism in the combination.

Macrocyclic lactones (MLs) are widely used in the treatment of humans, livestock and domestic animals infected with helminths. Eprinomectin (EPM) and ivermectin (IVM) are MLs with marked antiparasitic activity (Yilmaz et al., 2019). Both compounds are chemically related belonging to the avermectin subfamily. They target the gamma-aminobutyric acid (GABA) receptors and the glutamate-gated chloride ion channels (GluCl) and interfere with neurotransmission (Abongwa et al., 2017) (revised by Choudhary et al., 2022). This disturbance induces neuronal membrane hyperpolarization, paralysis, and ultimately the death of the parasite (Geary et al., 1993; Yates et al., 2003). Despite being very similar, they are used combined in commercial formulations or mixed by producers, under the assumption that their combined use would increase their efficacy and/or delay the spread of drug resistance. However, there is no data reported on the pharmacological evaluation of the EPM/IVM combination.

The lack of evidence for anthelmintic drug-drug interactions arises from the fact that clinical trials and field studies require a large number of animals, are costly, time-consuming, and difficult to replicate. *In vitro* studies with live parasites require either natural infections, which may contain mixed species populations, or artificial infections, which are costly and require *ex vivo* maintenance of parasites. Due to these limitations a complete drug-drug concentration matrix is not assessed in those studies, unlike the evaluation strategy that we propose in this study using the *Caenorhabditis elegans* model.

Many of the disadvantages mentioned above can be overcome by using the free-living nematode *C. elegans*. Among other advantages, *C. elegans* is easy and inexpensive to grow and maintain, it has a short life cycle with numerous progeny and it is amenable to genetics studies (Nigon and Félix, 2017). Despite not being a parasite, *C. elegans* is extremely useful for nematode parasitologists (Salinas and Risi, 2018; Hahnel et al., 2020). It belongs to nematode clade V, and therefore is a close relative to the major gastrointestinal parasitic nematodes of humans and livestock (e.g., *Haemonchus contortus* and *Cooperia* spp) (Blaxter et al., 1998). Indeed, *C. elegans* has been used in nematicide discovery projects (Burns et al., 2015; Partridge et al., 2018; Risi et al., 2019; Taki et al., 2021). Moreover, the mechanism of action and resistance to several anthelmintics has been elucidated using *C. elegans* (reviewed in Holden-Dye and Walker, 2014).

Being a key model for anthelmintic research, very few studies have used *C. elegans* to address drug-drug interaction between nematicides. Ding et al. (2017) reported that an optimal combination of four anthelmintics, which is more potent than any of them at lower concentrations than their EC50 values. Hu et al. (2010) showed that when Cry5B and nAChR agonists are combined, their activities are strongly synergistic. Despite these examples, there are no previous studies that have used the entire drug-drug concentration matrix to assess anthelmintic synergism.

In this study, we use a *C. elegans* high throughput infrared motility assay to address EPM/IVM interaction in matrix combinations (Simonetta and Golombek, 2007; Mathew et al., 2016). Our results indicates that there is neither synergism nor antagonism for EPM and IVM at any concentration ratio at any time, whichever the model used to analyze the data. Importantly, this study highlights several advantages of this model for pharmacodynamics and drug-drug interaction: simplicity, unlimited availability of nematodes, and the possibility to follow kinetics in a high throughput assay leading to high reproducibility.

## Materials and methods

### Chemicals

Technical grade eprinomectin (EPM) and ivermectin (IVM) were obtained from Compañía Cibeles S.A. (Montevideo, Uruguay). EPM and IVM were prepared at a 10 mM stock concentration in DMSO (41,640, Sigma-Aldrich) and stored at −20°C.

### *C. elegans* strains and culture methods

The *C. elegans* wild-type strain Bristol N2 (N2) and *Escherichia coli* OP50 strain were obtained from the *Caenorhabditis* Genomics Center (CGC, Minneapolis, MN, United States). Worms were grown on Nematode Growth Media (NGM) agar plates seeded with *E. coli* OP50 as a source of food, and maintained under standard conditions at 20°C. The N2 strain was cultured and maintained according to previously described procedures (Brenner, 1974; Stiernagle, 2006). Worm populations were synchronized at the stage of L1 and used in all assays at L4.

### Drug combination assay

L4 *C. elegans* worms were removed from culture plates and washed three times with K saline (NaCl 51 mM, KCl 32 mM) by centrifugation at 1,000 *g*. Worms were plated in 96-well microtiter plates (Costar 3,590). Approximately 80 worms per well were seeded in 60 µl of K saline containing 0.015% BSA. Compound dilution series in DMSO (0.5%) were added into the wells in a 6 × 6 matrix array. The matrix array included a control group (DMSO 0.5% without compounds), each drug alone at 5 concentrations (0.1; 0.2; 0.4; 0.8; 1.6 µM in 0.5% DMSO) and the 25 concentration combinations of both compounds. For each experiment replicas were performed.

Motility was measured using the infrared tracking device WMicrotrackerTM ONE (PhylumTech, Santa Fe, Argentina). The method used to assess motility is described in detail in (Simonetta and Golombek, 2007). Briefly, the system detects motility through the interference to an array of infrared light microbeams, caused by worm movement. The readout motility is count events (interruption of the beam by worm movement) per unit of time (5 min).

**FIGURE 1:**
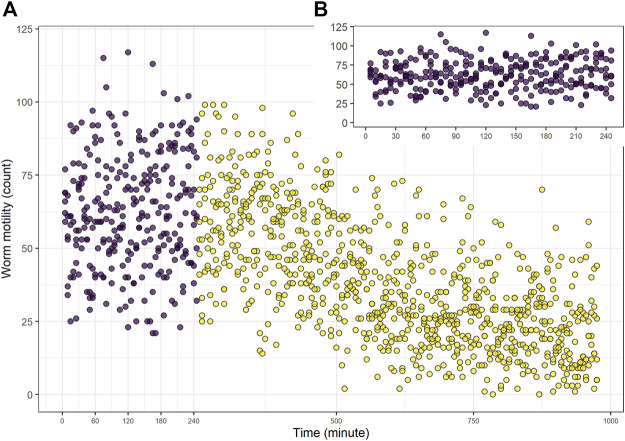
Control group worm motility. Control group (vehicle alone, DMSO 0.5%). Worm motility counts per unit of time (5 min) was measured for 980 min **(A)** Complete assay. Purple dots correspond to the data considered in subsequent dose-response assays (the first 240 min). **(B)** Inset time from 0 to 240 min only. The data shows a representative experiment.

The motility using WMicrotrackerTM ONE (Santa Fe, Argentina) was measured for 980 min (Figure 1A). We considered 240 min the optimal time to evaluate the drug effect, since the basal movement in the control group was constant during this time, but decreased markedly after 5 h (Figure 1B).

### Dose-response analysis

Worm motility of each well at 30, 60, 90, 120, 150, 180, 210, and 240 min was normalized to the median of the control wells to calculate the motility inhibition values (%). For synergy analysis, motility inhibition from replica wells was averaged and analyzed using the web-based tool SynergyFinder (Zheng et al., 2022).

The data were analyzed using a four-parameter log-logistic curve (sigmoidal shape) with variable slope and half-maximal inhibitory concentration (IC50) (Murado et al., 2002). The dose-response of a single drug and drug-drug interaction were visualized by pair heatmap and 3D surface motility inhibition [±95% confidence intervals (CI)].

Additionally, relative inhibition (RI) was determined considering the ratio of the area under the dose-response curve (adjusted by a four-parameter logistic model), with respect to the maximum area that a drug can reach in the same dose range.

### Drug synergy analysis

Data were analyzed using SynergyFinder Plus software (Zheng et al., 2022) considering four reference drug-drug interaction models: Highest Single Agent (HSA), Bliss Independence (Bliss), Loewe model (Loewe) and Zero Interaction Potency (ZIP). The models make different assumptions regarding the expected effect. In summary, the degree of synergy is quantified as the excess over the maximum single drug response (HSA); the multiplicative effect of single drugs as if they act independently (Bliss), the expected response corresponding to an additive effect as if the single drugs were the same compound (Loewe), or the expected response corresponding to the effect as if the single drugs did not affect the potency of each other (ZIP model) (Tang et al., 2015; Lanevski et al., 2020; Zheng et al., 2022). The drug-drug interaction models were visualized by pair heatmap and 3D surface of Synergy Score (±95% confidence intervals (CI)) and the average synergy score value in the whole combination matrix was determined. Synergy Score (SS) > 5 was categorized as strong synergy, whilst <5 was categorized as strong antagonism, according to (Yadav et al., 2015).

To determine the sensitivity of the drug pair, we used the combination sensitivity score (CSS) model that calculates the relative inhibition of a drug combination based on the area under the log10 scaled dose-response curves at the IC50 doses of the constituent drugs (Malyutina et al., 2019).

To visualize the maximal synergy and sensitivity scores we performed two-dimensional plots of these scores for all the analyzed models, as suggested by heng et al. (Malyutina et al., 2019; Zheng et al., 2022).

### Statistical analysis

Statistical analyses were performed with RStudio (Version 2022.2.3.492) (RStudio Team, 2022). The dose-response and synergy analysis were calculated using the package SynergyFinder of the R software fitting a dose-response sigmoid curve (four-parameter logistic). CSS of a drug combination was calculated such that each of the compounds (background drug) at a fixed concentration (its relative IC50) and the other (foreground drug) at varying concentrations resulting in two CSS values, which are then averaged (Malyutina et al., 2019; Zheng et al., 2022). Graphs were plotted using ggplot2 (Wickham, 2016)in R (Version 4.2.1) (R Core Team, 2022). All compound combination ratio drugs were randomized using random codes on plates to prevent experimental bias.

## Results

### Worm motility for single-drug treatment

**FIGURE 2:**
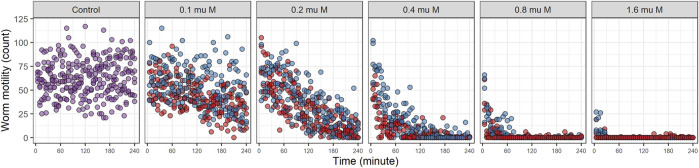
Locomotive activity for drugs alone. Worm motility over time was registered for 240 min. Different drug dose was used (range of 0.1–1.6 µM). Eprinomectin is depicted in red, Ivermectin in blue and Control in purple.

Locomotive activity for worms treated with EPM, IVM (range of 0.1–1.6 µM) and control group (without drugs) are shown in Figure 2. The activity distribution trends were similar for EPM and IVM, both drugs clearly differ from the control group (Figure 2). Inhibition of worm motility was observed at all concentrations examined. At the highest concentration (1.6 µM) the maximal effect (100% inhibition) was observed in the first 30 min. Below 1 µM inhibition with EPN was faster than with IVM. Sporadic movement was observed. We verified that worms in wells in which no motility was registered at the end of the experiment were dead (did not exhibit movement or touch response on plates).

### Dose-response curve

**FIGURE 3:**
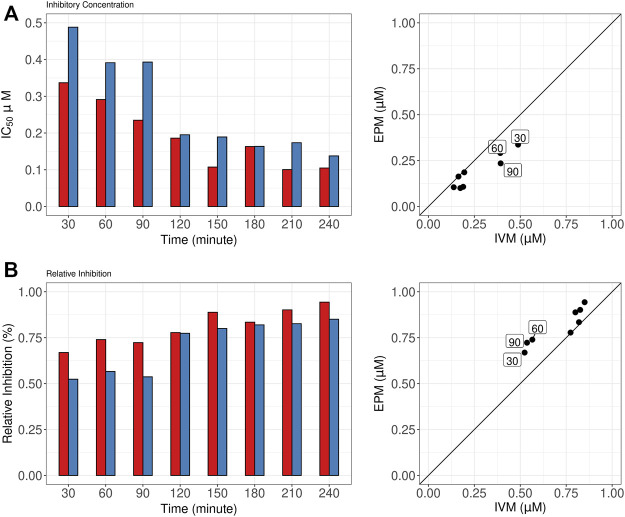
Half-maximal inhibitory concentration (IC50) and relative inhibition (RI) for single drugs*.* **(A)**. Eprinomectin (EPM) and Ivermectin (IVM) IC50 (left) and IC50 EPM/IVM ratios (right). 30–240 min exposure times are shown **(B)**. EPM and IVM RI (left) and EPM/IVM RI ratios. 30–240 min exposure time are shown. The squares highlight the higher potency of EPM than IVM at 30, 60, and 90 min.

Figure 3 shows the variation in IC50 (Panel A) and RI (Panel B) to single drugs in the dose-response model from 30 to 240 min. For both drugs potency (IC50) and RI increased as a function of exposure time reaching similar maximum values after 120–150 min. Nevertheless, in the first 90 min of the assay, EPM showed higher potency (lower IC50 values) than IVM (Panel A, left graph). The IC50 EPM/IVM ratios at 30, 60, and 90 min were 0.69, 0.74, and 0.59, respectively (Panel A, right graph). Similarly, EPM showed higher RI values than IVM at these times (Panel B, left graph). The EPM/IVM ratios 1.27, 1.30, and 1.34 at 30, 60, and 90 min, respectively (Panel B, right graph).

### Synergy effect and combination sensitivity score for EPM and IVM

**FIGURE 4:**
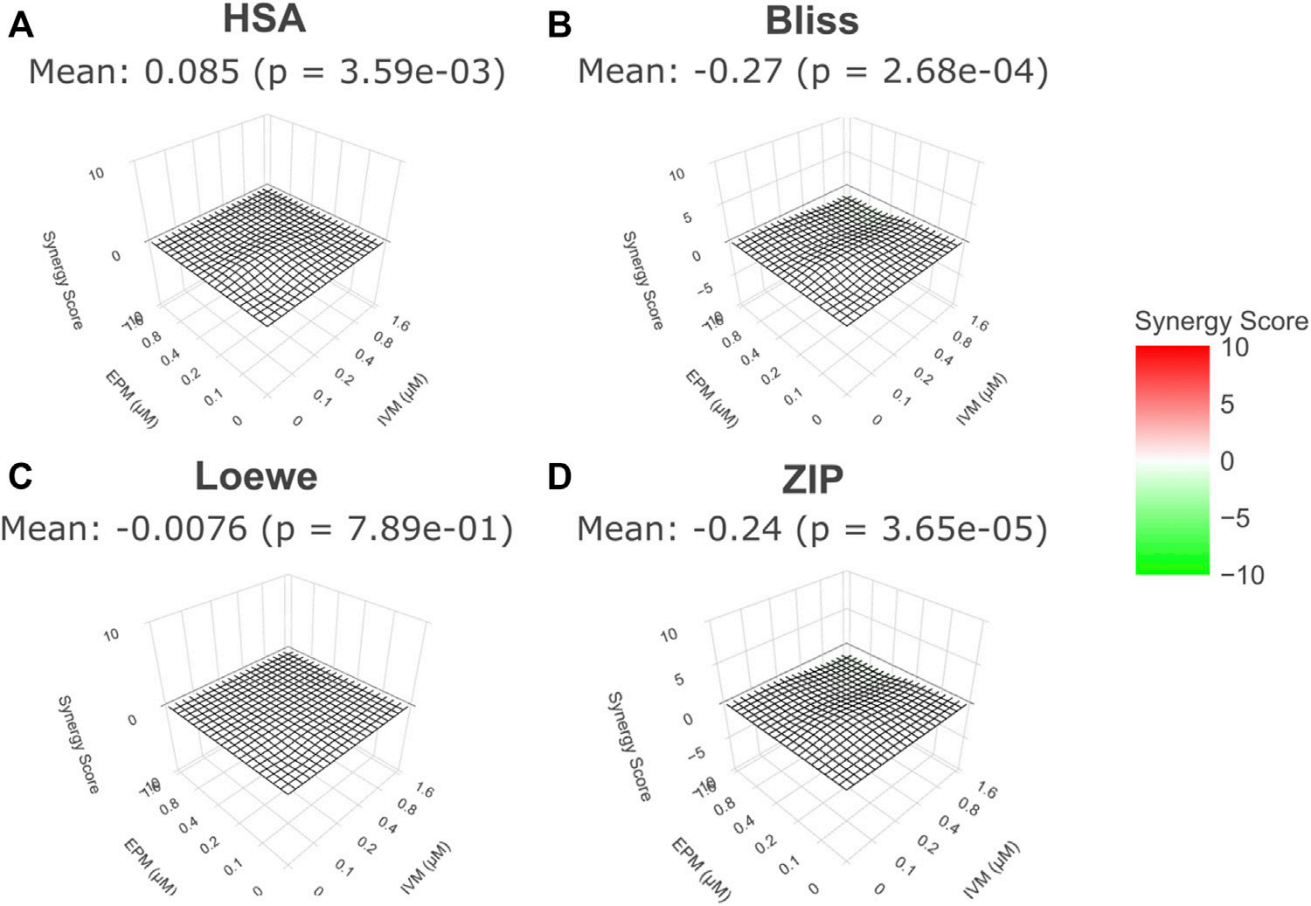
3D surface of Synergy Scores for EPM and IVM drug-drug interaction*.* For all models, EPM and IVM interaction landscapes are shown in 3D **(A)**. Highest Single Agent model (HAS) **(B)**. Bliss Independence model (BLISS) **(C)**. Loewe model (LOEWE) **(D)**. Zero Interaction Potency model (ZIP). 90 min exposure time is shown in all cases. The mean value of Synergy Score is indicated in the SD surface (*p* value compared to 0% inhibition). No significant synergy (red) or antagonism (green) between EPM and IVM at all concentrations.

3D surface of Synergy Scores (SS) for EPM and IVM drug-drug interaction for the four models (HSA, Bliss, Loewe and ZIP) were derived from the data. The different models used to study drug-drug interactions make different assumptions and define synergy and antagonism differently (BLISS, 1939; Loewe, 1953; Berenbaum, 1989; Yadav et al., 2015). In any case, none of the models suggest synergy or antagonism between both drugs. The 3D surface of SS at minute 90 is shown in Figure 4, but the same pattern was observed at all timepoints (Supplementary Figures S1–S8). In this representation, synergy has a positive value and is depicted in red and green has a negative value and is depicted in green. Values above 5 or below −5 are considered the threshold to define a synergic or antagonist effect, respectively (Malyutina et al., 2019).

**FIGURE 5:**
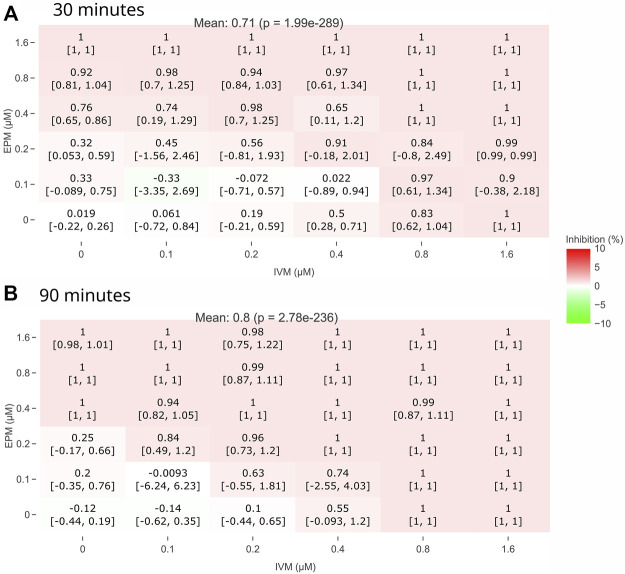
Heatmaps of the dose-response matrix for EPM and IVM drug-drug interaction*.* Exposure time at 30 min **(A)** and 90 min **(B)**. Mean value of the dose-response matrix is indicated at the top (*p* value compared to 0% inhibition). No significant change in inhibition (red or green) between EPM and IVM at all concentrations.

Pair heatmaps of the dose-response matrix for EPM and IVM at 30 and 90 min are shown in Figure 5. The same pattern was observed at all times (Supplementary Figures S1–S8). These times were selected since they showed minimal and maximal Combination Sensitivity Score (CSS). The heatmaps revealed that there was an additive effect, but neither significant synergism nor antagonism was observed.

**FIGURE 6:**
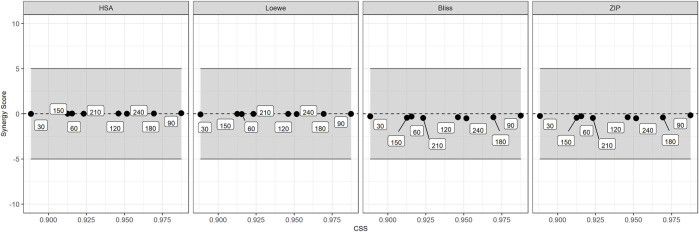
Synergy Scores (SS) and the overall Combination Sensitivity Score (CSS) for the combination EPM/IVM matrix*.* Each panel represents a different reference model of Synergy Scoring models with different assumptions regarding the expected effect (HSA = Highest Single Agent; BLISS = Bliss Independence; LOEWE = Loewe model and ZIP = Zero Interaction Potency). The gray zone indicates additive effect (Malyutina et al., 2019). Squares indicate the different times.

SS and the CSS for the entire combination matrix EPM/IVM are shown in Figure 6. The SS values were lower than ±0.5 for all experimental times, revealing the absence of synergism or antagonism, since the threshold for drug-drug interaction is ±5% (Malyutina et al., 2019). Indeed, all mean SS values were close to zero in the four reference models (HSA, Loewe, Bliss and ZIP), indicating additive interactions in all the concentration range. The CSS in motility reduction at different times varied marginally (from 0.02 to 0.1). The lowest and highest CSS were observed at exposure times of 30 and 90 min, respectively.

## Discussion

In this study we used the free-living nematode *C. elegans*, the simplest animal model, to study drug-drug interaction of widely used anthelmintics against parasitic nematodes. *C. elegans* is a firmly established model, but it has not been used before for thorough drug-drug interactions. Our results showed that this animal model is very useful since it can easily provide a large amount of data, which would be very difficult to obtain with nematode parasites *in vitro*, or with animal infections *in vivo*.

The results showed that there is a clear dose effect for both drugs. A time-dependent response was observed for both drugs (i.e., the longer the time the greater the effect, as evidenced by the lower IC50 with increased time). Furthermore, at the concentration range used, the effect was not reversible: once the drug paralyzed the worm, motility did not recover (the sporadic motility measured can be considered as a minor intrinsic variability, as observed for the control group). This is consistent with original reports for the IVM mode of action, which described its effect as persistent paralysis on nematode pharyngeal (Brownlee et al., 1997; Pemberton et al., 2001) and body wall musculature (Kass et al., 1980, 1982).

The exposure time of parasites to the active drug concentration is a critical factor that affects the efficacy and/or persistence of activity of most anthelmintics used in ruminants and determines the anthelmintic activity (Lanusse et al., 2018). The *C. elegans* motility assay is ideally suited to study time exposure to nematicides *in vitro*. Our results clearly reinforce the concept that nematicide activity increases with time at a constant concentration. The comparison of the time-dependent IC50 for EPM and IVM showed that EPM is slightly more potent than IVM, particularly before the first 2 h. This was confirmed by the relative inhibition, which was also greater for EPM than for IVM. These results contribute to visualize the importance of adequate anthelmintic dosage and exposure time, which may be relevant factors in delaying anthelmintic resistance.

*C. elegans* was also found valuable to reliably study drug-drug interactions *in vitro*. Taken together, the CSS and SS data show that EPM and IVM act through an additive mode. The lack of synergism supports previous assumptions that both macrocyclic lactones are supposed to act on the same target on nematodes (Martin et al., 2021; Choudhary et al., 2022). The structure of EPM and IVM would suggest that they act through the same mechanism (Lespine et al., 2012; David et al., 2016).

In view that the combined action of EPM and IVM is not superior to their individual actions, our results discouraged the combined use of these drugs, which are sometimes used not only by farmers but also in commercial formulations. Furthermore, our results suggest that EPM would be a better active principle than IVM for nematodes. EPM has a better dose-response time and efficacy than IVM (this study), less prolonged residual concentration in milk (Imperiale et al., 2021), and both drugs have similar toxicity to animals (Salman et al., 2022).

A note of caution is needed. There are limitations to this kind of study. *C. elegans* is a free-living nematode, and therefore valuable results should be further addressed using parasitic nematodes. Secondly, it is a reductionist approach that does not consider what happens within the host, in which there are other relevant interactions. Indeed, whether the results can be extended to infer what happens within the host is not simple. PKPD studies for each drug and for a selected combination complemented by efficacy studies would help to validate the model. Another approach that could contribute to reliable inferences is to determine the concentrations of drugs inside and outside the worm *in vitro* and *in vivo* conditions, in the worm environment. However, this reductionist approach has the enormous advantage to evaluate numerous different combinations to select a promising specific combination.

Drug combinations are widely used in parasitology to delay the development of drug resistance. However, in contrast to the field of antibiotics or antitumor drugs, there is a lack of models to assess synergy or antagonism in helminths. Our results show that *C. elegans* is a valuable nematode model for addressing antihelmintic drug-drug interactions *in vitro*, which is important before using drug combinations without field studies or proper assessment. Due to their simplicity, reproducibility and low cost, it could be a reference model to rationalize the use of different active principles, particularly against nematodes.

## Data Availability

The original contributions presented in the study are included in the article/Supplementary Materials, further inquiries can be directed to the corresponding authors.
