# Supplementary material for: Caenorhabditis elegans as a valuable model for the study of anthelmintic pharmacodynamics and drug-drug interactions: The case of ivermectin and eprinomectin
Source: Front Pharmacol. 2022 Oct 19;13:984905. doi: 10.3389/fphar.2022.984905 (PMC9627147; doi:10.3389/fphar.2022.984905)
Supplement: Supplementary file 1 [file DataSheet1.docx]

Supplementary Material

## Supplementary Figures

**
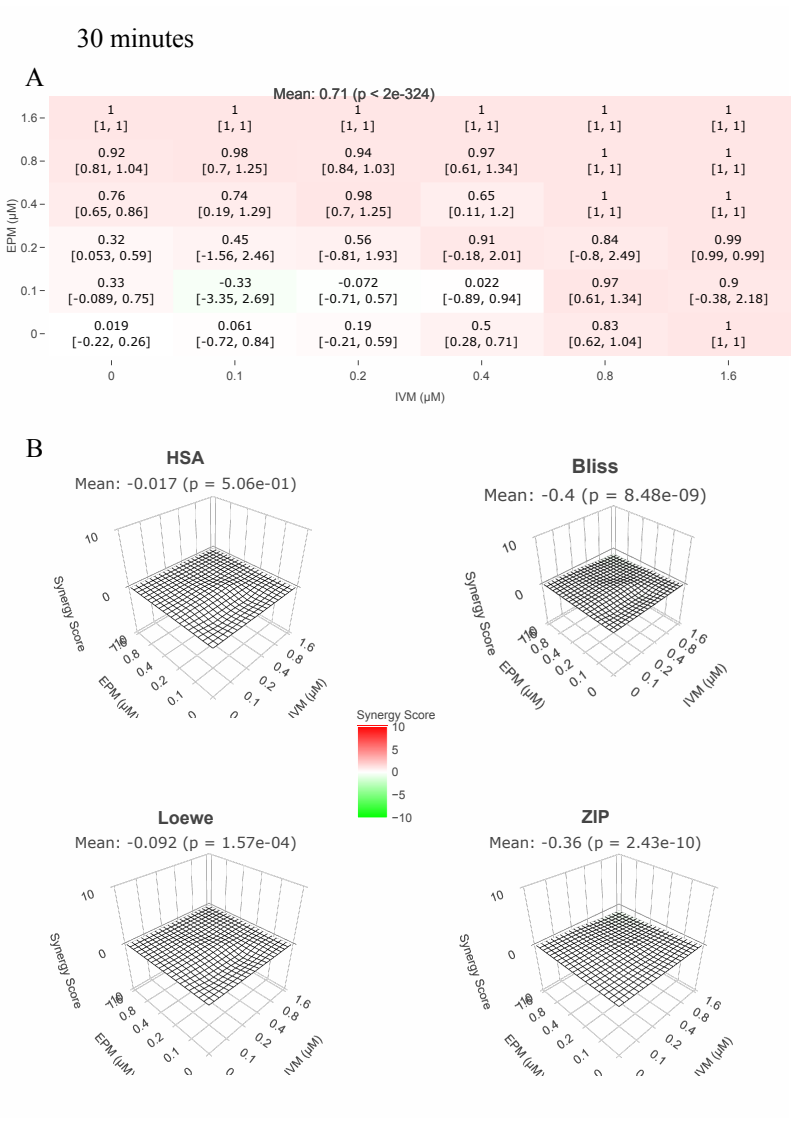
Supplemental Figure 1. EPM and IVM drug-drug interaction at 30 minutes. A.** Heatmaps of the dose-response matrix. **B.** 3D surface of Synergy. The Highest Single Agent model (HAS), Bliss Independence model (BLISS), Loewe model (LOEWE) and Zero Interaction Potency model (ZIP) were used. Neither synergic not antagonist effect was observed at any concentration ratio. No significant synergy (red) or antagonism (green) between EPM and IVM at all concentrations.

**
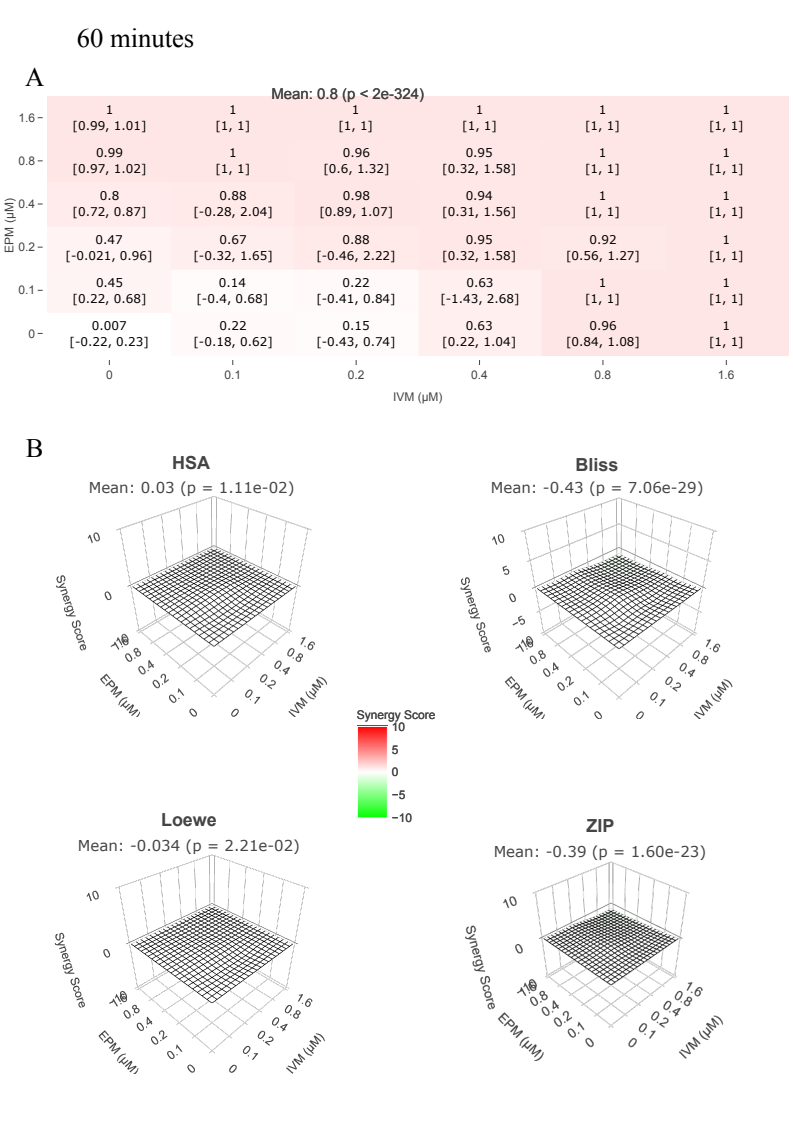
Supplemental Figure 2. EPM and IVM drug-drug interaction at 60 minutes. A.** Heatmaps of the dose-response matrix. **B.** 3D surface of Synergy. The Highest Single Agent model (HAS), Bliss Independence model (BLISS), Loewe model (LOEWE) and Zero Interaction Potency model (ZIP) were used. Neither synergic not antagonist effect was observed at any concentration ratio. No significant synergy (red) or antagonism (green) between EPM and IVM at all concentrations.

**
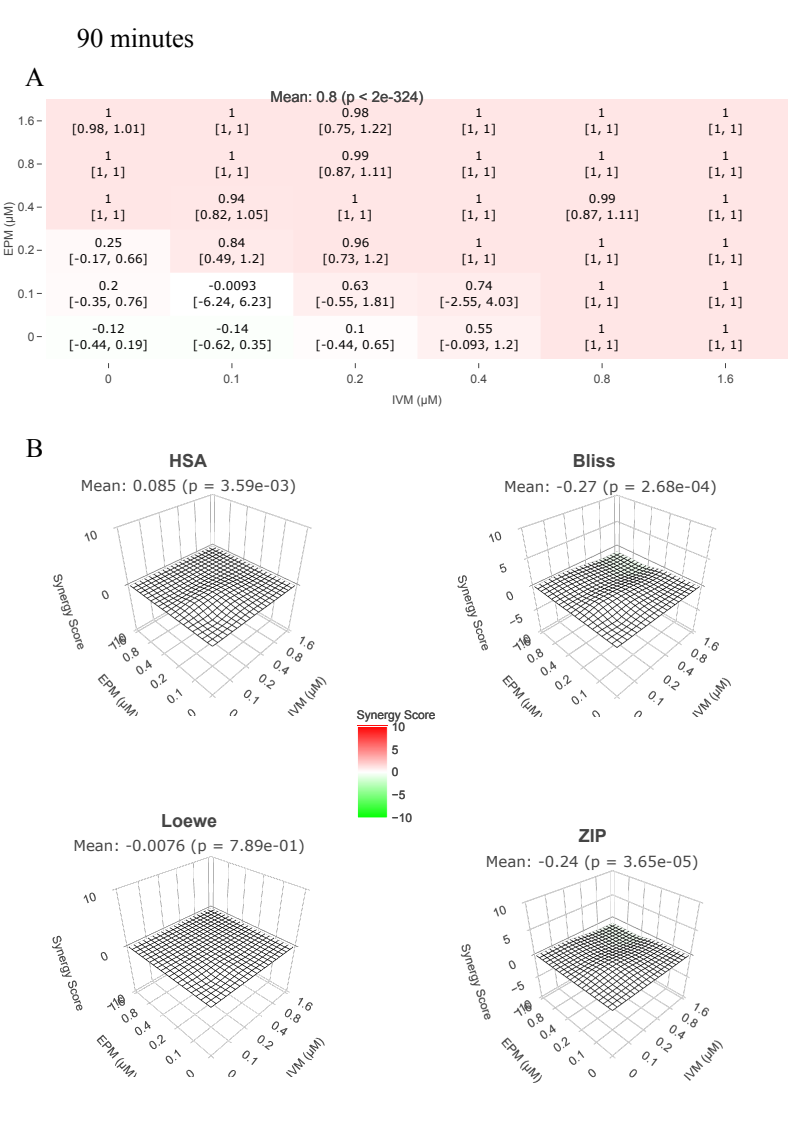
Supplemental Figure 3. EPM and IVM drug-drug interaction at 90 minutes. A.** Heatmaps of the dose-response matrix. **B.** 3D surface of Synergy. The Highest Single Agent model (HAS), Bliss Independence model (BLISS), Loewe model (LOEWE) and Zero Interaction Potency model (ZIP) were used. Neither synergic not antagonist effect was observed at any concentration ratio. No significant synergy (red) or antagonism (green) between EPM and IVM at all concentrations.

**
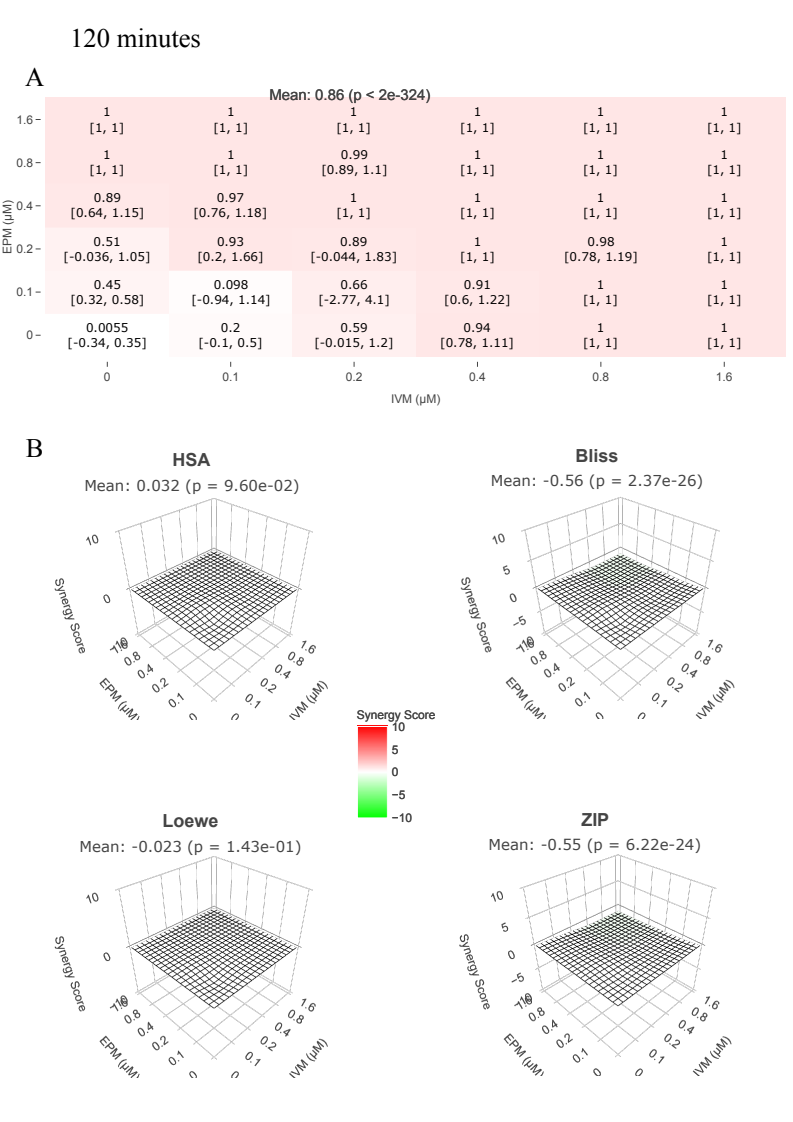
Supplemental Figure 4. EPM and IVM drug-drug interaction at 120 minutes. A.** Heatmaps of the dose-response matrix. **B.** 3D surface of Synergy. The Highest Single Agent model (HAS), Bliss Independence model (BLISS), Loewe model (LOEWE) and Zero Interaction Potency model (ZIP) were used. Neither synergic not antagonist effect was observed at any concentration ratio. No significant synergy (red) or antagonism (green) between EPM and IVM at all concentrations.

**
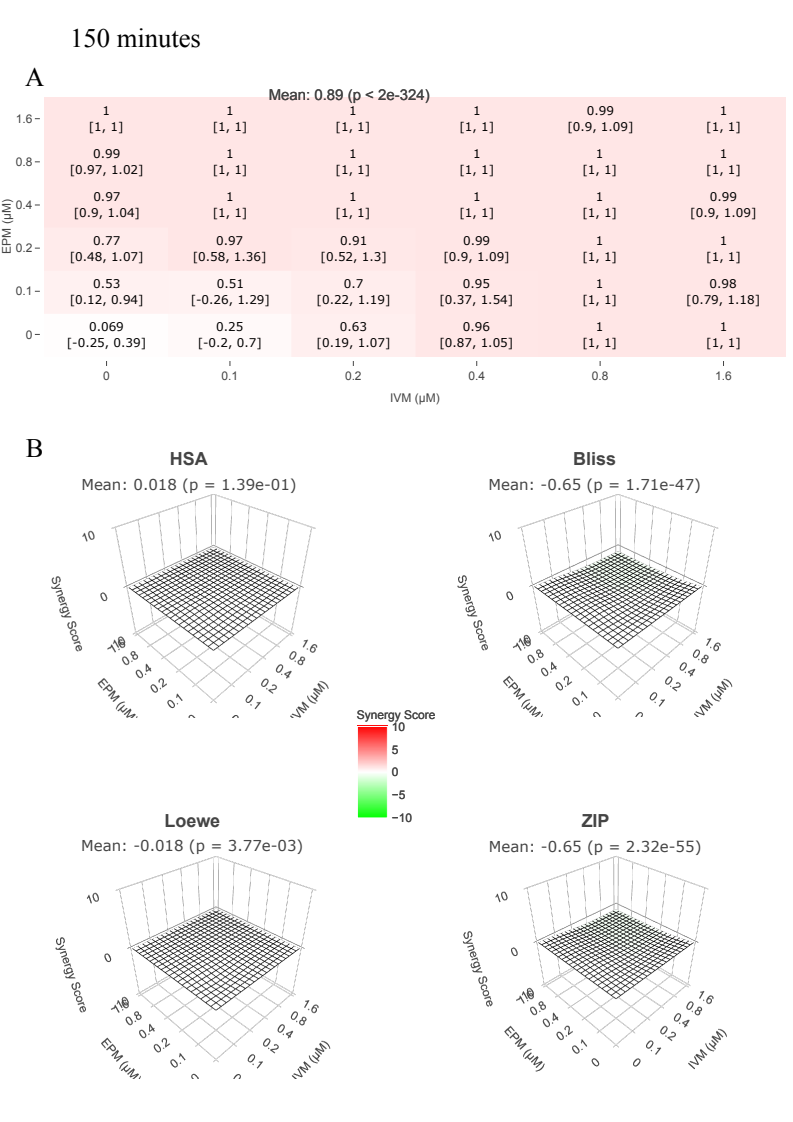
Supplemental Figure 5. EPM and IVM drug-drug interaction at 150 minutes. A.** Heatmaps of the dose-response matrix. **B.** 3D surface of Synergy. The Highest Single Agent model (HAS), Bliss Independence model (BLISS), Loewe model (LOEWE) and Zero Interaction Potency model (ZIP) were used. Neither synergic not antagonist effect was observed at any concentration ratio. No significant synergy (red) or antagonism (green) between EPM and IVM at all concentrations.


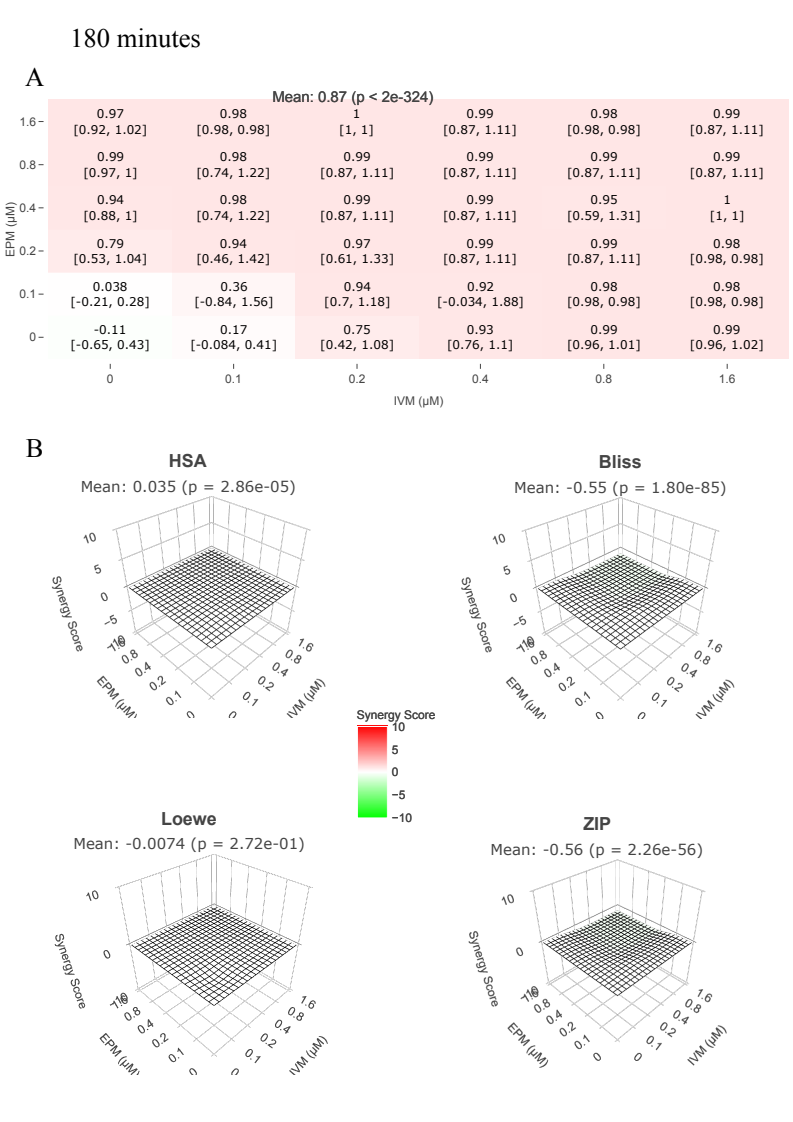
**Supplemental Figure 6. EPM and IVM drug-drug interaction at 180 minutes. A.** Heatmaps of the dose-response matrix. **B.** 3D surface of Synergy. The Highest Single Agent model (HAS), Bliss Independence model (BLISS), Loewe model (LOEWE) and Zero Interaction Potency model (ZIP) were used. Neither synergic not antagonist effect was observed at any concentration ratio. No significant synergy (red) or antagonism (green) between EPM and IVM at all concentrations.


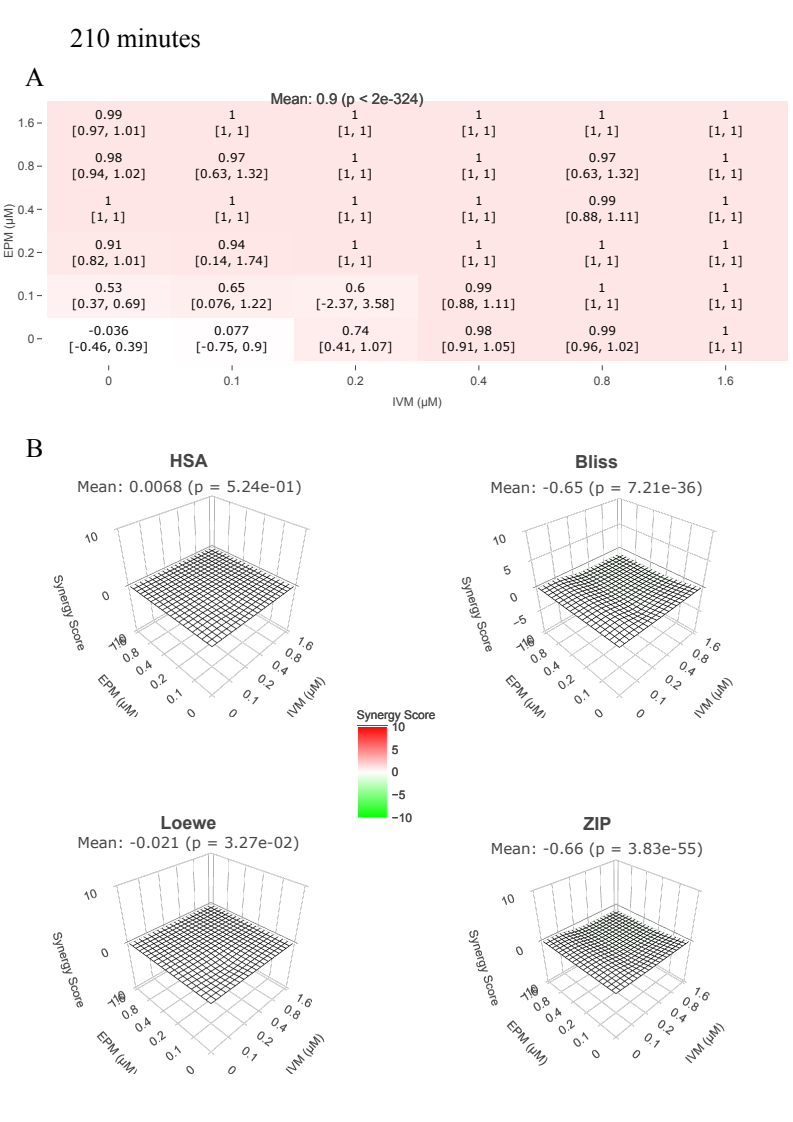
**Supplemental Figure 7. EPM and IVM drug-drug interaction at 210 minutes. A.** Heatmaps of the dose-response matrix. **B.** 3D surface of Synergy. The Highest Single Agent model (HAS), Bliss Independence model (BLISS), Loewe model (LOEWE) and Zero Interaction Potency model (ZIP) were used. Neither synergic not antagonist effect was observed at any concentration ratio. No significant synergy (red) or antagonism (green) between EPM and IVM at all concentrations.


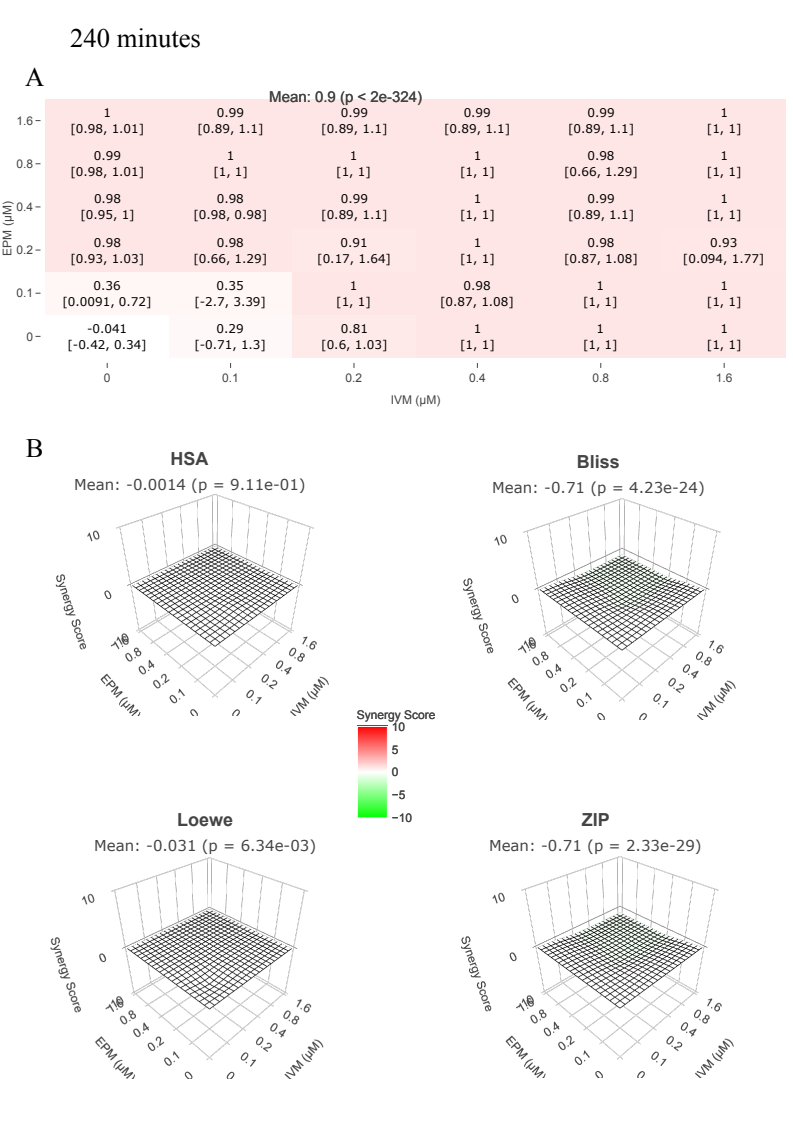
**Supplemental Figure 8. EPM and IVM drug-drug interaction at 240 minutes. A.** Heatmaps of the dose-response matrix. **B.** 3D surface of Synergy. The Highest Single Agent model (HAS), Bliss Independence model (BLISS), Loewe model (LOEWE) and Zero Interaction Potency model (ZIP) were used. Neither synergic not antagonist effect was observed at any concentration ratio.
